# Effects of Fucoxanthin on the Inhibition of Dexamethasone-Induced Skeletal Muscle Loss in Mice

**DOI:** 10.3390/nu13041079

**Published:** 2021-03-26

**Authors:** Maki Yoshikawa, Masashi Hosokawa, Kazuo Miyashita, Hoyoku Nishino, Takeshi Hashimoto

**Affiliations:** 1 Faculty of Sport and Health Sciences, Ritsumeikan University, 1-1-1 Nojihigashi, Kusatsu 525-8577, Japan; sh0047xv@ed.ritsumei.ac.jp; 2 Faculty of Fisheries Sciences, Hokkaido University, 3-1-1, Minato-cho, Hakodate 041-8611, Japan; hoso@fish.hokudai.ac.jp (M.H.); miyashitak@obihiro.ac.jp (K.M.); 3 Obihiro University of Agriculture and Veterinary Medicine, Nishi 2 Sen-11 Inabacho, Obihiro 080-0834, Japan; 4 Kyoto Prefecture University of Medicine, 465 Kajii-cho, Kawaramachi-Hirokoji, Kamigyo-ku, Kyoto 602-8566, Japan; hnishino@koto.kpu-m.ac.jp

**Keywords:** carotenoid, antioxidant, sarcopenic obesity

## Abstract

Fucoxanthin (Fx) has preventive effect against muscle atrophy and myotube loss in vitro, but it has not yet been examined in vivo. Therefore, we aimed to investigate the effect of Fx on dexamethasone (Dex)-induced muscle atrophy and fat mass in mice. ICR mice were fed with Fx diets from 2 weeks before Dex treatment to the end of the study. Muscle atrophy was induced in the mice by oral administration of Dex. Body weight was significantly lower by Dex treatment. Visceral fat mass in the Fx-treated group were significantly lower than those in the control group. The Dex-induced decrease in tibialis anterior muscle mass was ameliorated by Fx treatment. Fx treatment significantly attenuated muscle lipid peroxidation compared with the control and Dex-treated groups. The phosphorylation of AMPK was significantly higher in the Dex-treated group than in the control group. The expression of cytochrome c oxidase (COX) IV was significantly higher in the Fx-treated group than in the control group. These results suggest that Fx may be a beneficial material to prevent muscle atrophy in vivo, in addition to the effect of fat loss.

## 1. Introduction

Sarcopenia, which involves both muscle mass and strength, decreases with age and is associated with frailty and geriatric syndrome, leading to poor quality of life and loss of independence [1,2]. On the other hand, increasing body fat induces obesity, which causes secretion abnormalities of inflammatory cytokines, such as tumor necrosis factor alpha (TNFα) and insulin resistance [3]. Then, high levels of inflammatory cytokines and insulin resistance exaggerate sarcopenia by suppressing muscle protein synthesis [4]. Oxidative stress is considered a mechanism that links aging- and obesity-related inflammation and insulin resistance to sarcopenia. Oxidative stress causes damage to mitochondria and DNA, induces apoptosis, and causes muscle atrophy and loss of muscle cells [5]. Therefore, to eliminate and/or to suppress promotion of sarcopenia, it may be necessary to inhibit oxidative stress-induced muscle atrophy, in addition to fat loss. To date, some nutritional materials show preventive effects against obesity [6] or sarcopenia [7]. However, a few studies have investigated the dual effects of nutritional material on anti-obesity and antisarcopenia, which could sever the negative linkage between obesity and sarcopenia.

Fucoxanthin (Fx) is a carotenoid specifically produced by brown algae such as *Undaria pinnatifida* and diatoms such as *Phaeodactylum tricornutum*. Fx is metabolized into fucoxanthinol (FxOH) and amarouciaxanthin A in the body [8,9]. It is already known that Fx and its metabolites have various actions, such as antioxidant [10], anticancer [11], and anti-obesity activities. The anti-obesity effect of Fx and its metabolite have been extensively studied in cells and animals, and the effect and mechanism of action have been investigated. For instance, Fx suppresses the differentiation of preadipocytes into adipocytes [12]. FxOH increases lipolytic activity and reduces triacylglycerol levels in mature human adipocytes [13]. In addition, our recent study also confirmed that FxOH not only enhances lipolytic activity but also suppresses lipogenesis in 3T3-L1 adipocytes [14]. In an in vivo study, the administration of Fx suppressed the increase in white adipose tissue weight in mice [15]. Moreover, in human, Fx supplementation reduces body weight, BMI, and abdominal fat in adults with a body mass index (BMI) of greater than 25 and less than 30 kg/m^2^ [16]. Of note, our recent research clarified that FxOH suppresses hydrogen peroxide-induced muscle atrophy in myotubes, suggesting a potential dual effect against sarcopenia and obesity of Fx [14]. In this regard, in vivo studies to examine the effect of Fx on antisarcopenia might be warranted.

Animal models of skeletal muscle atrophy include unloading, fasting, denervation, and administration of glucocorticoids. In particular, the administration of high concentrations of dexamethasone (Dex) causes muscle protein degradation [17]. Glucocorticoid-induced proteolysis is primarily mediated by the ubiquitin proteasome system and the autophagy pathway. In particular, Atrogin-1 and MuRF1 are highly stimulated by glucocorticoid. In addition, Dex increases reactive oxygen species (ROS) production. In fact, the addition of Dex to L6 myotubes increased ROS production [18]. ROS are one of the factors that may play an important role in the induction of sarcopenia [19]. Age-related ROS overproduction causes oxidative damage to muscles and contributes to skeletal muscle atrophy [20]. On the other hand, a previous in vivo study showed that *Fructus Schisandrae* exerts an antioxidant effect related to muscle tissue protection against Dex-induced muscle atrophy in mice [17]. Quercetin glycosides suppress Dex-induced skeletal muscle atrophy in mice in addition to myotubes [21]. β-carotene suppresses muscle atrophy by reducing oxidative stress in mice [22]. These findings suggest that antioxidants have a protective effect against oxidative stress-induced muscle atrophy in vivo, which can be induced by the administration of oxidative stressors such as Dex. We hypothesized that Fx/FxOH, which are known to possess stronger antioxidant activity than other carotenoids [23], also have a preventive effect on oxidative stress-induced muscle atrophy in vivo, which may extend the anti-obesity effect of Fx. Thus, in this study, we investigated the effect of the administration of Fx on the inhibition of the Dex-induced decrease in muscle mass in adult mice.

## 2. Materials and Methods

### 2.1. Animal Care

The animal study was approved by the Committee on Animal Care at Ritsumeikan University (BKC2017-007). Five-week-old ICR male mice were obtained from Japan SLC, Inc., Shizuoka, Japan, and cared for according to the Guiding Principles for the Care and Use of Animals based on the Declaration of Helsinki. Mice were housed one mouse per cage under controlled conditions (12/12-h light/dark cycle, a temperature-controlled environment (23 ± 1 °C)). The mice had free access to drinking water and were fed a control diet MF (Oriental Yeast CO., Ltd., Tokyo, Japan) [24]. After a 12-week acclimation period, the mice were split up into three groups: the intact vehicle control (control, *n* = 7), the Dex-treated group (Dex, *n* = 7), or the Fx- and Dex-treated group (Fx-Dex, *n* = 8).

### 2.2. The Administration of Fucoxanthin and Dexamethasone

In the Dex and Fx-Dex groups, muscle atrophy was induced by oral administration of dissolved dexamethasone (Sigma-Aldrich, St. Louis, MO, USA) in sterile water (1 mg/L) for 13 days. The mice in the Fx-Dex group were fed with Fx (0.2%) mixed MF diets from 2 weeks before administration of Dex to the end of the study [15]. Fx was obtained by Oryza Oil & Fat Chemical Co., Ltd. (Aichi, Japan). The test food was provided in powder food containing FucoRex™-5 (Oryza Oil & Fat Chemical Co. Ltd., Aichi, Japan, with 5% fucoxanthin). After the experimental period, body weight was measured after fasting for 12 h. After sacrifice, the visceral fat, tibialis anterior, gastrocnemius, and soleus muscles were removed, weighed, and frozen in liquid nitrogen.

### 2.3. Malondialdehyde Assay

Frozen muscle tissue samples were ground to a powder using a bead pulverizing machine (Bio Medical Science Co., Ltd., Tokyo, Japan). Muscle tissue powder was dissolved in RIPA buffer (10 mM Tris-HCl (pH 7.4), 150 mM NaCl, 5 mM EDTA, 0.1% sodium dodecyl sulfate (SDS), 1% Triton, 1% Na deoxycholate) appended with phenylmethane sulfonyl fluoride (PMSF) and protease inhibitor cocktail (Sigma-Aldrich, USA). Samples were sonicated on ice and then centrifuged at 1600× *g* for 10 min at 4 °C. The supernatant was collected and protein concentration was analyzed with the Wako protein assay kit (FUJIFILM Wako Pure Chemical Corporation, Osaka, Japan), and samples were prepared as 2.0 mg/mL protein using 0.1% BSA/PBS. The levels of malondialdehyde (MDA), a marker of oxidative stress, in skeletal muscle were measured using the OxiSelect^TM^ MDA Adduct Competitive ELISA kit (Cell Biolabs, Inc., San Diego, CA, USA; STA-832) according to the manufacturer’s instructions.

### 2.4. Western Blot Analysis

Frozen muscle tissue samples were ground to a powder using a bead pulverizing machine (Bio Medical Science Co., Ltd.). Muscle tissue powder was dissolved in RIPA buffer appended with PMSF, protease inhibitor cocktail (Sigma-Aldrich, USA), phosphatase inhibitor cocktail, and PhosSTOP phosphatase inhibitor cocktail (Sigma-Aldrich, USA). Samples were incubated on ice for 1 h and then centrifuged at 15,000× *g* for 15 min at 4 °C. The supernatant was collected, and the protein concentration was analyzed with the Wako protein assay kit (FUJIFILM Wako Pure Chemical Corporation, Japan). Equal amounts of protein extracts (10–20 µg) were separated by 8–12% SDS-polyacrylamide gel electrophoresis at 30 mA for 2 h and transferred to a polyvinylidene fluoride membrane at 60 V for 2 h. After blocking for 30 min with Blocking One P (NACALAI TESQUE, INC, Kyoto, Japan) at room temperature, membranes were incubated with primary antibodies against mTOR (1:1000; Cell Signaling Technology, Danvers, MA, USA; #2983), phosphorylated-mTOR (1:1000; Cell Signaling Technology; #5536), p70S6K1 (1:1000; Cell Signaling Technology; #2708), phosphorylated-p70S6K1 (1:1000; Cell Signaling Technology; #9205), TRIM63 (MuRF1) (1:8000; Proteintech, Rosemont, IL, USA; 55456-1AP), AMP-activated protein kinase (AMPK) (1:1000; Cell Signaling Technology; #2793), phosphorylated-AMPK (Thr172) (1:1000; Cell Signaling Technology; #2535), COX IV (1:2000; Cell Signaling Technology; #4850), VDAC (1:1000; Cell Signaling Technology; #4866), LC3A/B (1:1000; Cell Signaling Technology; #12741), anti-SOD1 (1:1000; Sigma-Aldrich; SAB2500976), anti-SOD2 (1:1000; Sigma-Aldrich; SAB2102261), and glyceraldehyde-3-phosphate dehydrogenase (GAPDH) (1:10,000; Sigma-Aldrich; G9545). Immunoreactive proteins were incubated with anti-mouse IgG (1:10,000), anti-goat IgG (1:10,000), and anti-rabbit IgG (1:10,000) horseradish peroxidase (HRP)-linked whole antibody (Sigma-Aldrich, USA) to detect primary antibody binding. After washing three times in TBST for 10 min each, chemiluminescence quantification was performed using Luminata Forte Western HRP Substrate (Millipore Corporation, Billerica, MA, USA), followed by detection with FUSION FX (Vilber-Lourmat, Île-de-France, France). Band intensities was quantified by ImageJ software (National Institutes of Health, Bethesda, MD, USA) [24,25].

### 2.5. Statistical Analyses

All data are presented as the means ± S.D. Multiple comparison tests were performed with Dunnett’s method using 4-Step Excel statistics (OMS). A value of *p* < 0.05 indicated statistically significant.

## 3. Results and Discussion

### 3.1. The Administration of Fx May Reduce Atrophy Caused by Dex-Induced Oxidative Stress

**Figure 1:**
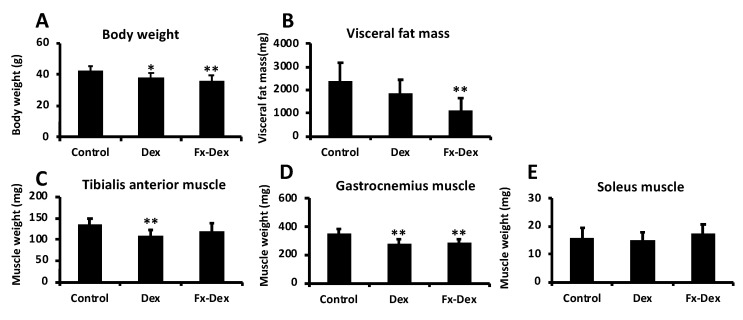
Fucoxanthin (Fx) protected against dexamethasone (Dex)-induced muscle atrophy in mice. The graph shows the body weight (**A**), visceral fat mass (**B**), tibialis anterior muscle weight (**C**), gastrocnemius muscle weight (**D**), and soleus muscle weight (**E**). Values represent the mean ± S.D. (*n* = 7–8). Significant differences were determined by Dunnett’s test (* *p* < 0.05, ** *p* < 0.01).

The present study examined whether the administration of Fx would suppress oxidative stress-induced atrophy in mice. We found that body weights in the Dex and Fx-Dex groups were significantly lower than that in the control group (Figure 1A). Furthermore, visceral fat mass in the Fx-Dex group was significantly lower than that in the control group (*p* < 0.01) (Figure 1B). Notably, as shown in Figure 1C, the tibialis anterior muscle mass in the Dex group was significantly lower than that in the control group (*p* < 0.01), whereas the administration of Fx inhibited the Dex-induced decreases in tibialis anterior muscle mass. The gastrocnemius muscle mass in the Dex and Fx-Dex groups were significantly lower than that in the control group (Figure 1D). There was no significant difference in the soleus muscle mass under all conditions (Figure 1E). These results suggest that Fx attenuates the Dex-induced decrease in tibialis anterior muscle mass and decreases fat mass.

Glucocorticoid is known to cause little or no atrophy in slow muscle, but it does cause muscle atrophy in fast muscle [26]. Fast muscles (e.g., tibialis anterior muscle) are more susceptible to glucocorticoid-induced muscle atrophy than slow muscle (e.g., soleus muscle) [27]. Glucocorticoid receptor is more highly expressed in fast muscles than in slow muscles [28]. Glucocorticoid, acting via glucocorticoid receptor, may regulate muscle protein mass through signals involved in various muscle proteolysis processes [29]. In the present study, administration of Dex to the tibialis anterior and gastrocnemius muscles, which include many fast-twitch muscles, decreased their muscle mass. However, Fx prevented the Dex-induced reduction in muscle mass in tibialis anterior muscle.

**Figure 2:**
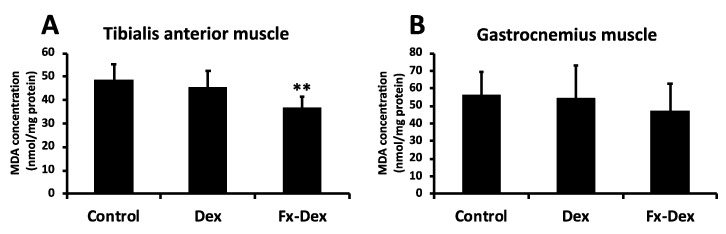
Fx reduced the levels of malondialdehyde (MDA) in tibialis anterior muscle. The graph shows the concentration of MDA in the tibialis anterior muscle (**A**) and gastrocnemius muscle (**B**). Values represent the mean ± S.D. (*n* = 7). Significant differences were determined by Dunnett’s test (** *p* < 0.01).

**Figure 3:**
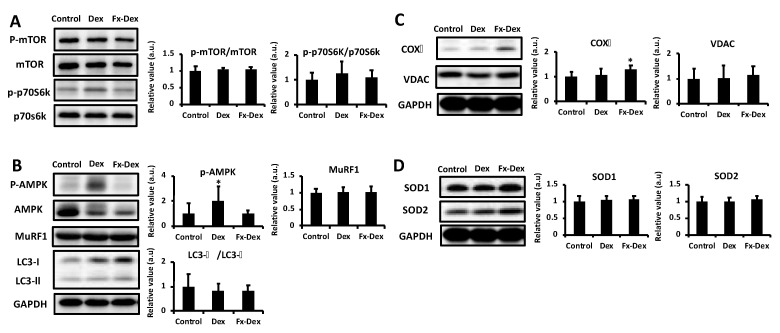
Fx inhibited Dex-induced phosphorylation of AMP-activated protein kinase (AMPK) and increased the expression of cytochrome c oxidase (COX) IV in tibialis anterior muscle. The graph shows the expression of protein synthesis-related proteins (**A**), degradation-related proteins (**B**), mitochondria-related proteins (**C**) and antioxidant enzymes (**D**). Values represent the mean ± S.D. (*n* = 6–7). All protein expression levels were normalized to the glyceraldehyde-3-phosphate dehydrogenase (GAPDH) levels. Protein is expressed relative to the value of the control protein levels. Significant differences were determined by Dunnett’s test (* *p* < 0.05).

**Figure 4:**
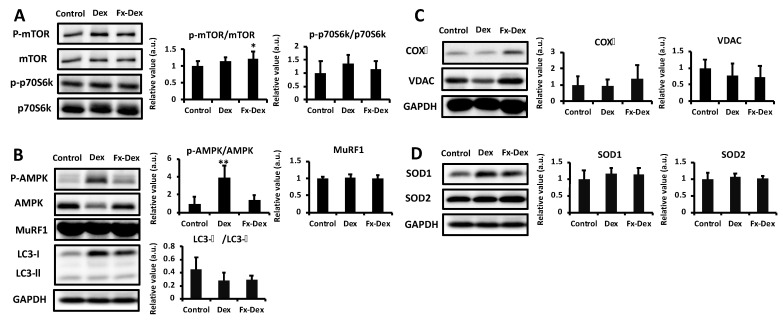
Fx inhibited Dex-induced phosphorylation of AMPK in gastrocnemius muscle. The graph shows the expression of protein synthesis-related proteins (**A**), degradation-related proteins (**B**), mitochondria-related proteins (**C**), and antioxidant enzymes (**D**). Values represent the mean ± S.D. (*n* = 5–7). All protein expression levels were normalized to the GAPDH levels. Protein is expressed relative to the value of the control protein levels. Significant differences were determined by Dunnett’s test (* *p* < 0.05, ** *p* < 0.01).

Because muscle weight changes by administration of Dex and Fx were observed in tibialis anterior and gastrocnemius muscles, we explored potential mechanisms underlying the changes in those muscles. Administration of glucocorticoid leads to oxidative stress and induces mitochondrial dysfunction in skeletal muscle [30]. Mitochondrial dysfunction leads to further ROS production and protein degradation. In this regard, inhibition of increased levels of lipid peroxidation in muscle tissue under oxidative stress and an increase in antioxidant enzymes are important in terms of muscle protection [17]. As shown in Figure 2A, muscle lipid peroxidation in the tibialis anterior muscle was significantly lower in the Fx-Dex group than in the control and Dex groups (*p* < 0.01). However, there was no significant difference in lipid peroxidation under all conditions in gastrocnemius muscle (Figure 2B). On the other hand, the expression of SOD1 and SOD2, which are antioxidant enzymes, was not changed in any group in either the tibialis anterior or gastrocnemius muscle (Figure 3D and Figure 4D). In the present study, lipid peroxidation levels in tibialis anterior muscles were suppressed by Fx treatment, but the oxidative stress defense system was not changed, suggesting a direct inhibitory effect of Fx on ROS [14]. Thus, Fx inhibits muscle atrophy through reduced oxidative stress in tibialis anterior muscle, although the mechanism is unclear.

### 3.2. Effects of Fx on Protein Expression Levels in the Tibialis Anterior and Gastrocnemius Muscles

The administration of Dex promotes proteolysis and inhibits protein synthesis [31] via glucocorticoid receptor and induces oxidative stress, which further leads to muscle atrophy. Based on the difference in the tibialis anterior and gastrocnemius muscle weights and MDA levels, the expression of muscle protein synthesis- or degradation-related proteins was analyzed in both the gastrocnemius and tibialis anterior muscles. Glucocorticoid-induced proteolysis is mainly mediated by the ubiquitin proteasome system and the autophagy pathway [32]. The ubiquitin proteasome pathway plays an important role in muscle degradation. In fact, the exposure of myotubes to Dex increases the expression of MuRF1 and decreases myosin heavy chain protein [33]. Moreover, Dex induces muscle atrophy via the induction of autophagy [34]. However, in this study, there were no significant differences in the expression level of MuRF1 or the LC3-II/LC3-I ratio under all groups in either the tibialis anterior or the gastrocnemius muscle (Figure 3B and Figure 4B). On the other hand, the phosphorylation of mTOR was significantly higher in the Fx-Dex group than in the control group in the gastrocnemius muscle. However, there were no significant differences in the phosphorylation of p70S6K among all groups in either the tibialis anterior or the gastrocnemius muscle (Figure 3A and Figure 4A). There is a possibility that Fx might not affect the proteolysis pathway. Nonetheless, the Fx-revealed potential of muscle protein synthesis (i.e., increased phosphorylation of mTOR) in gastrocnemius muscle should not be ignored, and further studies are needed.

Mitochondrial dysfunction increases AMP and ROS and activates AMPK, which suppresses protein synthesis via inhibition of mTOR signaling [35] and hence results in muscle atrophy [36]. In this study, phosphorylation of AMPK was significantly higher in the DEX group than in the control and Fx-Dex groups in both the tibialis anterior and gastrocnemius muscles (*p* < 0.01) (Figure 3B and Figure 4B). On the other hand, in the tibialis anterior muscle, the expression level of COX IV, an enzyme of the mitochondrial respiratory chain, was significantly higher in the Fx-Dex group than in the control group (Figure 3C). Dex induces mitochondrial dysfunction and decreases ATP production. Dex causes protein degradation and muscle atrophy by activating the AMPK/FOXO signaling pathway [36]. In this study, Fx suppressed the activation of AMPK and increased the expression of COX IV induced by Dex in tibialis anterior. However, there was no change in the expression level of muscle protein degradation-related proteins. Thus, the administration of Fx might inhibit muscle atrophy by improving mitochondrial dysfunction and subsequently suppressing ROS levels and AMPK activity.

In the present study, we found that fucoxanthin simultaneously inhibited muscle atrophy and induced fat loss in vivo. However, due to the lack of an experimental group with fucoxanthin alone or a weight matched control group, it is unclear whether any effects occur without Dex or weight loss. Future studies are necessary to examine the effect of fucoxanthin alone on muscle mass and fat accumulation using another model, such as an aged animal model, and determine whether fucoxanthin is beneficial not only in the individuals suffering from a deleterious condition but also in the healthy individuals.

To conclude, the addition of Fx reduced oxidative stress-induced muscle atrophy by Dex treatment, specifically in the tibialis anterior muscle. Although the precise mechanism should be further elucidated, Fx decreased ROS and increased the expression of mitochondria-related protein. There was also a signal that could positively regulate the balance between muscle protein synthesis and degradation, such as increased phosphorylation of mTOR and suppression of the AMPK activation. The results suggest that Fx may be a beneficial material to prevent Dex-induced muscle atrophy in vivo, in addition to the effect of fat loss.

## Data Availability

The datasets used and/or analyzed during in this study are available from the corresponding author on reasonable request.
